# Supplementary material for: Physics-informed convolutional neural networks for fluid flow through porous media
Source: Sci Rep. 2026 Jun 18;16:27879. doi: 10.1038/s41598-026-55389-w (PMC13547363; doi:10.1038/s41598-026-55389-w)
Supplement: Supplementary file 1 [file 41598_2026_55389_MOESM1_ESM.pdf]

Supplementary Information

Physics-informed convolutional neural networks for fluid flow through porous media

Rafał Topolnicki[1,2]**, Paweł Dłotko**[1]**, Maciej Matyka**[3,4]**,**

[1]Dioscuri Center in Topological Data Analysis, Institute of Mathematics, Polish Academy of Sciences, ul. Sniadeckich 8, 00-656 Warsaw, Poland

[2]Institute of Experimental Physics, University of Wrocław, pl. Maxa Borna 9, Wrocław 50-204, Poland

[3]Institute of Theoretical Physics, Faculty of Physics and Astronomy, University of Wrocław, pl. Maxa Borna 9, Wrocław 50-204, Poland

[4]Parallel and Distributed Systems Laboratory, Jožef Stefan Institute, Jamova cesta 39, Ljubljana 1000, Slovenia

Contents

S1 Error Bounds 2

S2 Training procedure details 3

S3 Loss function hyperparameters 5

S4 Fourier Neural Operators 5

S1 Error Bounds

To assess the robustness of the surrogate in the regimes highlighted by the referee, we complement global error metrics with a conditional uncertainty analysis based on the velocity magnitude $|v|$. In Fig. S1, we plot a two-dimensional histogram of $|v|_{\mathrm{pred}}$ versus $|v|_{\mathrm{LBM}}$, which shows both the concentration of samples around the ideal relation $y = x$ and the spread of the prediction error as a function of the predicted velocity magnitude. The purpose of estimating confidence bounds is to move beyond a single averaged score and quantify how predictive uncertainty changes across the state space, in particular in difficult low-porosity regimes where transport pathways become constricted and the flow becomes more heterogeneous. To this end, we define residuals

$$r = |v|_{\mathrm{LBM}} - |v|_{\mathrm{pred}}, \tag{S1}$$

and estimate conditional lower and upper quantiles of r within bins of the predicted velocity magnitude $|v|_{\mathrm{pred}}$. Denoting the conditional quantile function by $Q_\alpha(\cdot)$, the empirical estimates correspond to

$$q_\alpha(x) = Q_\alpha\big(r \,\big|\, |v|_{\mathrm{pred}} = x\big), \tag{S2}$$

where $\alpha \in \{0.05, 0.95\}$ in the present work. These quantiles define lower and upper bounds for the ground-truth velocity magnitude conditioned on the prediction:

$$\underline{v}(x) = x + q_{0.05}(x), \qquad \overline{v}(x) = x + q_{0.95}(x). \tag{S3}$$

The discrete estimates $\{(x_i, q_\alpha(x_i))\}$ obtained from binning are then converted into continuous functions using a shape-preserving piecewise cubic Hermite interpolant (PCHIP), [1, 2]. In practice, this corresponds to constructing a piecewise cubic function $s_\alpha(x)$ such that

$$s_\alpha(x_i) = q_\alpha(x_i), \quad i = 1, \ldots, N, \tag{S4}$$

with continuity of both $s_\alpha(x)$ and its first derivative, while preserving the local monotonicity of the data. The final confidence bounds are therefore given by

$$\underline{v}(x) = x + s_{0.05}(x), \qquad \overline{v}(x) = x + s_{0.95}(x). \tag{S5}$$

These discrete quantile estimates are then converted into continuous lower and upper bound functions using a shape-preserving piecewise cubic Hermite interpolant, PCHIP, [1, 2]. In practice, this interpolation constructs a cubic polynomial within each bin interval while enforcing continuity of both the function and its first derivative across bin boundaries. Unlike standard cubic splines, which may introduce non-physical oscillations or overshoots in regions with sparse or rapidly changing data, PCHIP explicitly preserves the local monotonicity and shape of the underlying quantile estimates. As a result, the interpolated bounds remain consistent with the empirical trends observed in the binned data, providing smooth yet faithful approximations of the conditional quantile curves without introducing artificial extrema or distortions.

The resulting bounds are shown globally as a band around the diagonal in Fig. S1, and spatially in Fig. S2, where the lower and upper confidence limits are evaluated pointwise as functions of the predicted velocity magnitude $|v|_{\mathrm{pred}}$. The multi-panel visualization provides additional insight into the spatial structure of the uncertainty: regions of high velocity, typically corresponding to preferential flow channels, remain tightly constrained, whereas low-velocity regions near solid boundaries and in poorly connected pore spaces exhibit a visibly wider confidence envelope. These regions are associated with reduced porosity and proximity to the percolation threshold, where the flow becomes more sensitive to small geometric variations. The predicted fields remain consistent with the topology and spatial organization of the LBM solution, and the ground-

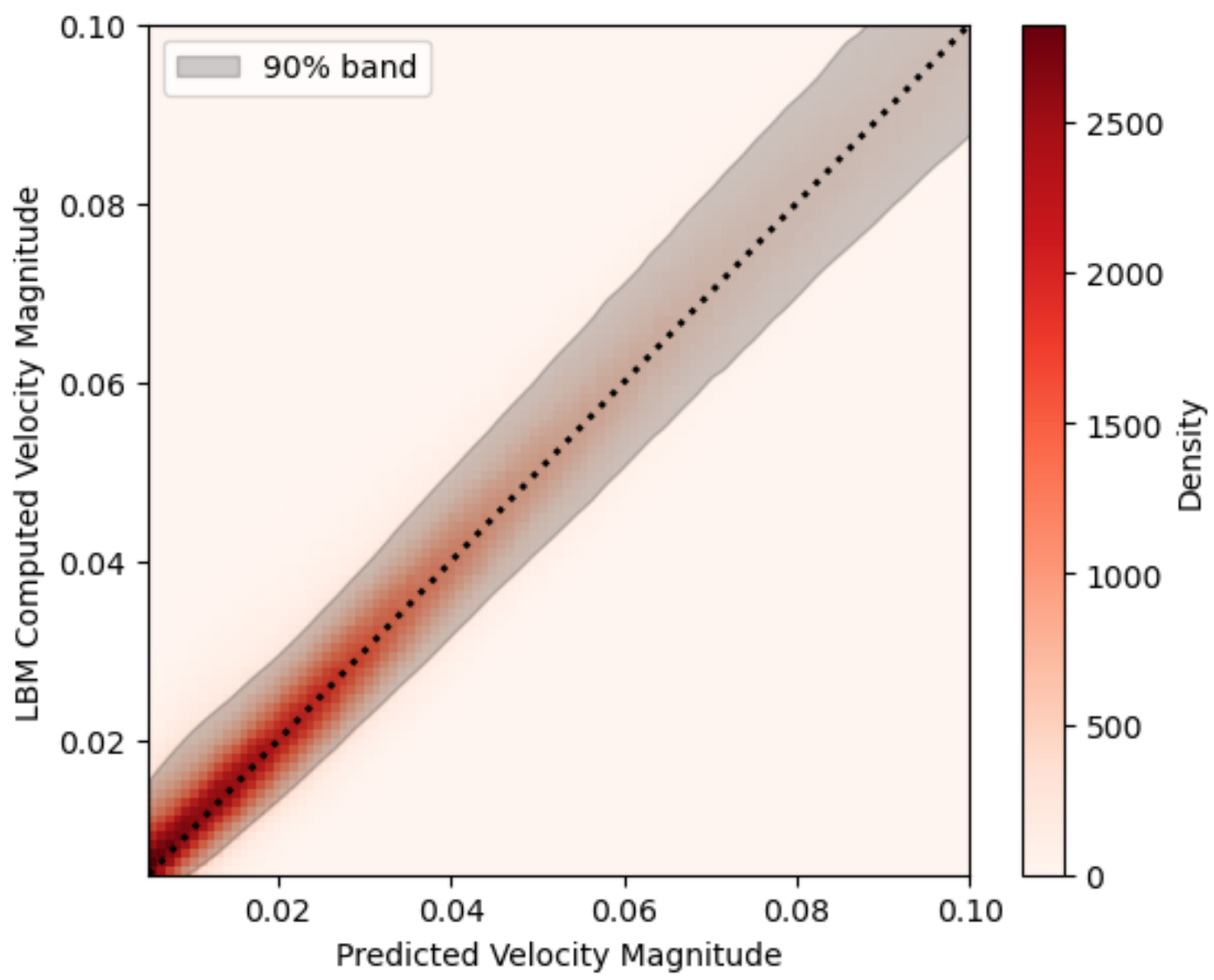

Figure S1: Two-dimensional histogram of ground-truth (LBM-computed) velocity magnitude $|v|_{\mathrm{LBM}}$ versus predicted velocity magnitude $|v|_{\mathrm{pred}}$. The color scale represents the density of samples. The dashed line indicates the ideal relation $|v|_{\mathrm{LBM}} = |v|_{\mathrm{pred}}$, while the shaded region denotes the estimated 90% conditional confidence band for $|v|_{\mathrm{LBM}}$ given $|v|_{\mathrm{pred}}$, obtained from empirical quantiles of the residuals $r = |v|_{\mathrm{LBM}} - |v|_{\mathrm{pred}}$. The narrow band in the high-density region indicates strong agreement between predictions and ground truth for moderate velocities, whereas the widening of the band at higher velocities reflects increased uncertainty in more challenging flow regimes.

truth solution is, in most regions, contained within the estimated bounds. This indicates that the model retains meaningful predictive capability while appropriately reflecting increased uncertainty in more complex flow configurations.

Overall, this conditional quantile-based analysis provides a more detailed characterization of model performance, highlighting how predictive reliability varies across the domain and offering spatially resolved bounds on the expected prediction error, rather than relying solely on global summary metrics.

The same uncertainty quantification framework can be applied independently to the individual components of the velocity vector. In this case, the analysis is performed for each component (e.g., v_x and v_y) by constructing conditional confidence intervals for the ground-truth component given its predicted value. This involves defining residuals $r = v_{\mathrm{LBM}} - v_{\mathrm{pred}}$ for each component and estimating conditional quantiles of r within bins of the predicted component. This extension allows for component-wise uncertainty characterization, capturing potential anisotropies in the prediction error while preserving the local structure of the flow field. In practice, the results are qualitatively consistent with the magnitude-based analysis, while providing additional directional insight into the model performance.

S2 Training procedure details

Training was performed on a workstation equipped with an NVIDIA GeForce RTX 4090 GPU with 24 GB of VRAM and an AMD Ryzen 9 5950X CPU. The dataset was randomly split into training, validation, and test subsets containing 70%, 15%, and 15% of the samples, respectively. A batch size of 32 was used in all experiments. During training, the loss was monitored on the validation set, and early stopping was applied with a patience of 20 epochs. The total number of epochs varied between architectures, but training typically

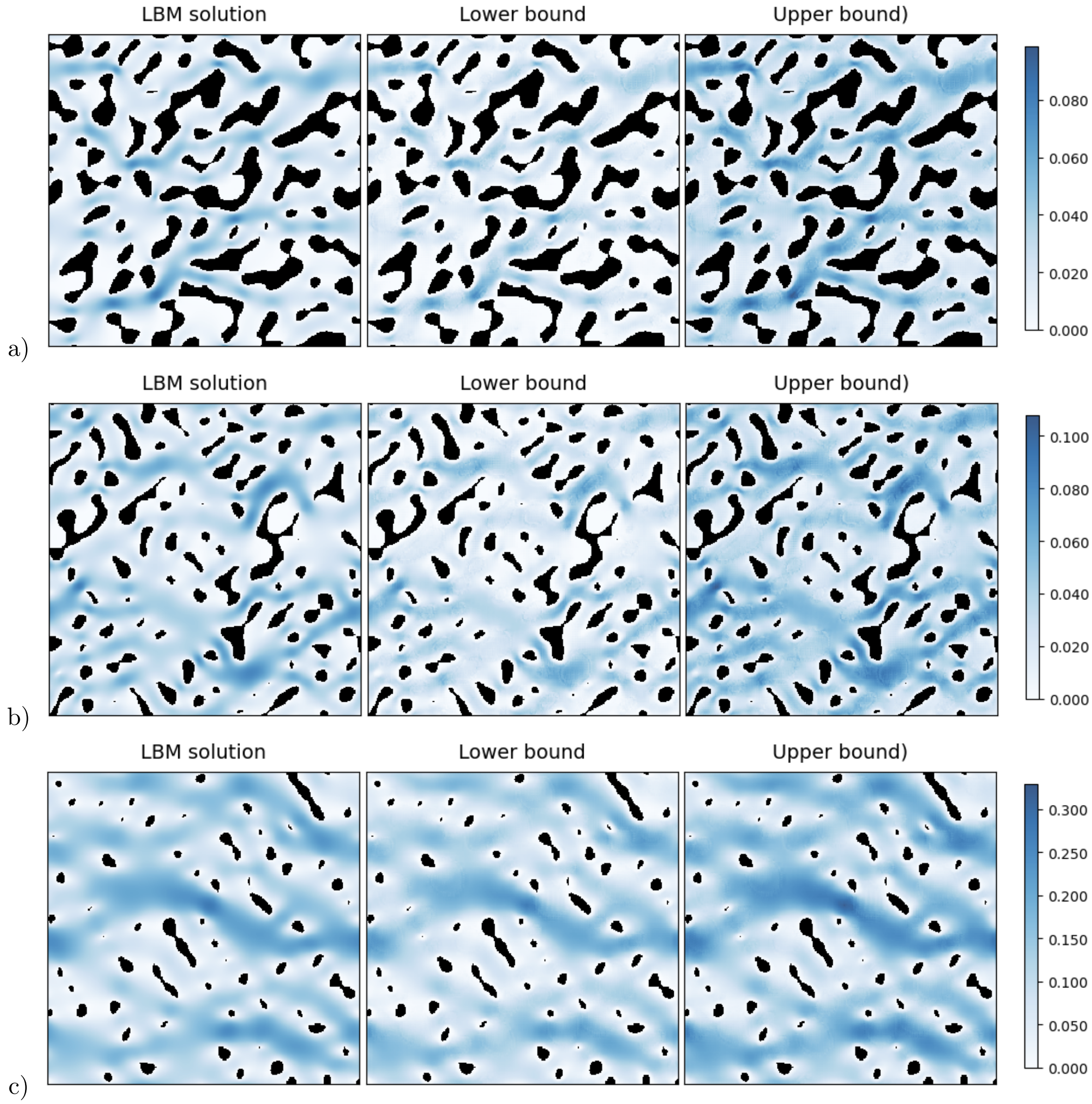

Figure S2: Spatial visualization of conditional confidence bounds for the velocity magnitude $|v|$. The left panel shows the ground-truth LBM solution, while the central and right panels display the estimated lower and upper confidence bounds, respectively, obtained from the conditional quantile model. Solid regions correspond to the pore structure. The bounds are evaluated pointwise as functions of the predicted velocity magnitude $|v|_{\text{pred}}$, resulting in spatially varying uncertainty estimates. Systems have porosities: a) 0.713, b) 0.817, c) 0.931.

required approximately 250 epochs. Optimization was performed using the Adam algorithm with an initial learning rate of 5×10^{-4}. The learning rate was updated using a step scheduler with step size 10 and decay factor `gamma=0.8`.

We found that the most stable convergence was obtained using a two-stage training procedure. In the first stage, the model was trained with the obstacle loss enabled and the remaining physics-based terms disabled, i.e. with $\alpha = 5$ and $\beta = \gamma = \delta = 0$. In the second stage, training was resumed from the weights obtained in the first stage using the full loss function with the final hyperparameter values $\alpha = 5, \beta = 1, \gamma = 0.1$ and $\delta = 0.01$.

S3 Loss function hyperparameters

The loss function introduced in Eq. (11) of the manuscript contains four hyperparameters: α, β, γ, and δ, which control the relative importance of different physical constraints. The terms weighted by α and β act locally, enforcing agreement with the reference velocity field and local physical constraints such as zero velocity inside obstacles and divergence-free flow. In contrast, the terms weighted by γ and δ operate at a global level, enforcing periodic boundary conditions and consistency of derived macroscopic quantities, such as tortuosity.

These parameters can therefore be interpreted as balancing local fidelity against global physical consistency. While their values are selected through standard hyperparameter tuning, they also provide a degree of flexibility, allowing the user to emphasize specific physical properties depending on the application.

To further investigate their influence, we performed additional experiments in which each hyperparameter was varied over approximately two orders of magnitude, while the others were kept fixed. The tested values were: $\alpha = 1, 5, 10$, $\beta = 0.1, 1, 10$, $\gamma = 0.01, 0.1, 1$, and $\delta = 0.001, 0.01, 0.1$, where the middle values correspond to those used in the main manuscript. Each experiment was repeated three times with different data splits, and median values are reported.

Table S1 summarizes the results. The table is organized in blocks corresponding to the parameter being varied, with the associated loss term highlighted for clarity. Increasing a given hyperparameter consistently reduces the corresponding component of the loss function, as expected. However, this does not necessarily translate into a reduction of the overall mean squared error (MSE), indicating non-trivial interactions between the different loss terms.

In many cases, adding additional loss components improves the overall prediction accuracy, which may be attributed to improved optimization and avoidance of local minima. Notably, the parameter set used in the main manuscript ($\alpha = 5$, $\beta = 1$, $\gamma = 0.1$, $\delta = 0.01$) corresponds to one of the lowest MSE values observed.

The interplay between local and global terms is not trivial. For example, introducing the global periodicity term ($\gamma > 0$) reduces the MSE, even though it primarily enforces a global constraint. Adding the tortuosity term ($\delta > 0$) slightly increases local error metrics but significantly improves the accuracy of predicted macroscopic quantities.

It is worth noting that the same set of hyperparameters, i.e. $\alpha = 5$, $\beta = 1$, $\gamma = 0.1$, and $\delta = 0.01$, was also used in the Fourier Neural Operator experiments described in Section S4. A similar trend is observed in that case: the inclusion of additional loss terms leads to a consistent improvement in all evaluated metrics. This indicates that the beneficial effect of the proposed loss formulation is not specific to convolutional architectures, but extends to operator-learning approaches as well.

Overall, these results indicate that the loss function components interact in a coupled manner, and that the choice of hyperparameters reflects a trade-off between different physical properties. The selected parameter values represent a balanced compromise between local accuracy and global physical consistency.

S4 Fourier Neural Operators

In addition to convolutional neural networks, we explored the use of Fourier Neural Operators (FNOs) as an alternative model class. FNOs are a recently proposed class of neural operators designed to learn mappings between function spaces, rather than between discrete images [3, 4]. They achieve this by performing global convolutions in the Fourier domain, enabling efficient modeling of long-range spatial dependencies. This makes them particularly appealing for problems governed by partial differential equations, such as fluid flow in porous media.

In our implementation, the FNO model operates on regular grids and consists of a sequence of spectral

Table S1: Performance metrics for models trained with different values of loss-function hyperparameters. All models use the same ResNet-101 architecture. Median values over three independent runs with different train/validation/test splits are reported. The table illustrates how individual loss components and prediction accuracy respond to changes in the weighting coefficients.

α	β	γ	δ	$\mathcal{L}_{\text{vel}}$	$\mathcal{L}_{\text{inside}}$	$\mathcal{L}_{\text{div}}$	$\mathcal{L}_{\text{periodic}}$	$\mathcal{L}_{\text{tort}}$	R^2_τ
1	0	0	0	8.79E-05	1.58E-05	4.15E-06	1.04E-04	1.73E-03	0.757
5	0	0	0	3.68E-05	4.43E-06	6.41E-06	3.34E-05	6.85E-04	0.901
10	0	0	0	6.61E-05	2.60E-06	1.28E-05	6.26E-05	2.86E-03	0.581
5	0.1	0	0	2.14E-05	1.40E-06	3.53E-06	2.32E-05	9.23E-04	0.859
5	1	0	0	2.00E-05	1.45E-06	2.00E-06	2.30E-05	1.09E-03	0.833
5	10	0	0	2.08E-05	1.70E-06	7.51E-07	2.16E-05	1.52E-03	0.768
5	1	0.01	0	2.02E-05	1.56E-06	1.90E-06	2.25E-05	7.12E-04	0.891
5	1	0.1	0	1.79E-05	1.42E-06	1.84E-06	1.86E-05	8.71E-04	0.873
5	1	1	0	1.71E-05	1.57E-06	1.80E-06	9.84E-06	1.52E-03	0.787
5	1	0.1	0.001	2.52E-05	2.26E-06	3.39E-06	2.50E-05	3.92E-04	0.943
5	1	0.1	0.01	2.07E-05	1.85E-06	2.09E-06	2.22E-05	1.24E-04	0.983
5	1	0.1	0.1	2.67E-05	2.15E-06	2.59E-06	2.63E-05	9.58E-05	0.986

Table S2: Comparison between the best-performing CNN architecture (ResNet101, see Table 2 in the manuscript) and the Fourier Neural Operator (FNO) models. Two FNO variants are considered: one trained with a simplified loss function and one trained using the full loss formulation adopted for CNNs. In all FNO cases, $\alpha = 5$.

	velocity	tortuosity			permeability		
	RMSE	RMSE	MAPE	R^2	RMSE	MAPE	R^2
ResNet101	5.10E-03	1.13E-02	0.6	0.983	8.69E+02	4.8	0.997
FNO $\beta = 0, \gamma = 0, \delta = 0$	5.52E-03	2.71E-02	1.37	0.897	1.26E+03	5.49	0.993
FNO $\beta = 1, \gamma = 0.1, \delta = 0.01$	5.48E-03	2.27E-02	0.98	0.928	1.31E+03	5.85	0.992

convolution layers interleaved with pointwise transformations. The input is augmented with spatial coordinate information, allowing the network to learn position-dependent mappings. The model was trained to predict the two components of the velocity field directly, using the same dataset and train–validation split as for the CNN-based models. After preliminary exploration of model capacity, we selected a configuration with 16 retained Fourier modes per spatial dimension, a channel width of 256, and 4 stacked spectral layers, which provided the best performance among the tested variants.

Two versions of the FNO model were trained. The first one employed a simplified loss function, corresponding to Eqs. (11–13) in the manuscript, with $\alpha = 5$ and $\beta = \gamma = \delta = 0$. The second version used the full loss formulation introduced for the CNN models, with $\beta = 1$, $\gamma = 0.1$, and $\delta = 0.01$. This allows us to assess whether the additional physics-informed terms in the loss function provide consistent benefits across different model architectures.

The results are summarized in Table S2. The FNO model achieves predictive performance of the same order of magnitude as the CNN-based approach, particularly for the velocity field reconstruction. More importantly, the inclusion of the additional loss terms leads to a systematic improvement across all evaluated metrics. In particular, the tortuosity prediction error decreases and the coefficient of determination R^2 increases when the full loss formulation is used. A similar trend is observed for permeability.

These observations indicate that the proposed loss function contributes to improved model performance independently of the underlying architecture. In particular, the gains obtained by incorporating the additional loss terms are not specific to convolutional networks, but extend to operator-learning approaches such as FNO. This suggests that the loss formulation plays a key role in guiding the model towards physically consistent predictions.

References

[1] Fritsch, F.N., Carlson, R.E., 1980. Monotone Piecewise Cubic Interpolation. SIAM Journal on Numerical Analysis 17, 238–246. 10.1137/0717021.

[2] Fritsch, F.N., Butland, J., 1984. A Method for Constructing Local Monotone Piecewise Cubic Interpolants. SIAM Journal on Scientific and Statistical Computing 5, 300–304. 10.1137/0905021.

[3] Li, Z., Kovachki, N., Azizzadenesheli, K., Liu, B., Bhattacharya, K., Stuart, A., Anandkumar, A., 2021. Fourier Neural Operator for Parametric Partial Differential Equations. International Conference on Learning Representations (ICLR).

[4] Kovachki, N., Li, Z., Azizzadenesheli, K., Liu, B., Bhattacharya, K., Stuart, A., Anandkumar, A., 2023. Neural Operator: Learning Maps Between Function Spaces. Journal of Machine Learning Research 24, 1–97.
